# Pneumocystis Pneumonia in a Non-HIV Patient With Advanced Liver Cirrhosis and No History of Immunosuppression: A Case Report

**DOI:** 10.7759/cureus.86557

**Published:** 2025-06-22

**Authors:** Yuta Isomura, Yuichiro Ogura, Hiroyuki Tamiya

**Affiliations:** 1 Respiratory Medicine, Tokushima Prefectural Miyoshi Hospital, Miyoshi, JPN

**Keywords:** immunocompromised host, liver cirrhosis, opportunistic infection, pneumocystis jirovecii, pneumocystis pneumonia

## Abstract

Pneumocystis pneumonia (PCP) is a critical opportunistic infection, typically seen in immunocompromised individuals. While its association with HIV is well known, there is a growing recognition of PCP in non-HIV patients, often linked to immunosuppressive therapy. However, cases of PCP in patients with liver cirrhosis and without iatrogenic immunosuppression remain underreported. We describe the case of a 72-year-old male patient with advanced liver cirrhosis (Child-Pugh score 12) secondary to metabolic dysfunction-associated steatohepatitis. He presented with fever and cough and was diagnosed with PCP based on characteristic imaging findings, elevated serum β-D-glucan, and a positive polymerase chain reaction test for *Pneumocystis jirovecii* in his sputum. Notably, he had no history of immunosuppressant use or HIV infection. Despite initial improvement in his respiratory condition with trimethoprim-sulfamethoxazole and corticosteroids, his hospital course was complicated by subsequent *Candida* bloodstream infection, ultimately leading to his death. This case highlights that advanced liver cirrhosis alone can constitute a significant risk factor for PCP and underscores the poor prognosis often associated with this condition. It emphasizes the importance of considering PCP in the differential diagnosis for patients with liver cirrhosis presenting with respiratory failure.

## Introduction

Pneumocystis pneumonia (PCP) is a potentially life-threatening infection that primarily occurs in immunocompromised patients. While PCP initially gained attention with the rise of human immunodeficiency virus (HIV)-infected individuals, the increasing incidence of PCP in non-HIV-infected patients undergoing immunosuppressive therapy (non-HIV-PCP) now represents a significant clinical challenge [1].

The primary risk factor for non-HIV-PCP is the use of immunosuppressants, including glucocorticoids. These medications are utilized in various clinical scenarios, such as the treatment of hematological malignancies, following hematopoietic stem cell transplantation, for collagen diseases, and to prevent rejection after organ transplantation [1]. Patients with liver cirrhosis are also considered at risk for PCP, particularly when receiving immunosuppressive therapy after liver transplantation.

On the other hand, liver cirrhosis itself is known to cause immune dysfunction [2]. Therefore, it is plausible that liver cirrhosis alone could be a risk factor for developing PCP, even in the absence of iatrogenic immunosuppression. However, reports focusing on this specific association are limited. Here, we report a case of non-HIV-PCP in a patient with liver cirrhosis who had no prior history of immunosuppressant use. Discussion with previous reports suggests an association of immunosuppressive status due to cirrhosis alone.

## Case presentation

A 72-year-old man presented to the emergency department with a chief complaint of fever and a cough that had persisted for two to three days. His past medical history was significant for hypertension, well-controlled diabetes mellitus, and liver cirrhosis related to metabolic dysfunction-associated steatohepatitis. He had previously undergone endoscopic treatment for esophageal variceal bleeding secondary to his liver cirrhosis. He had no history of using immunosuppressive drugs and had not been previously diagnosed with any immunodeficiency.

On arrival, his vital signs were as follows: body temperature 37.5°C, pulse rate 102 beats/min, blood pressure 154/72 mmHg, respiratory rate 26 breaths/min, and SpO_2_ 96% while receiving 3 L/min of oxygen via nasal cannula. Physical examination revealed icteric sclerae, abdominal distension, and lower extremity edema. Chest auscultation identified fine crackles in both lower lung fields. Laboratory tests showed elevated levels of lactate dehydrogenase (LDH), C-reactive protein (CRP), and Krebs von den Lungen-6 (KL-6) (Table 1). Additionally, hyperbilirubinemia and hypoalbuminemia were noted, consistent with his known liver cirrhosis.

**Table 1:** Laboratory data Upper limit for PT% not available in our system. Alb: albumin, ALP: alkaline phosphatase, ALT: alanine aminotransferase, ANA: anti-nuclear antibody, ANCA: anti-neutrophil cytoplasmic antibodies, APTT: activated partial thromboplastin, AST: aspartate aminotransferase, BNP: brain natriuretic peptides, BUN: blood urea nitrogen, CCP: cyclic citrullinated peptide, Cl: chloride, CMV: cytomegalovirus, Cre: creatinine, CRP: C-reactive protein, Hb: hemoglobin, IgG: immunoglobulin G, K: potassium, KL-6: Krebs von den Lungen 6, LDH: lactate dehydrogenase, Lympho: lymphocyte, Na: sodium, Neutro: neutrophil, MPO: myeloperoxidase, Pj: *Pneumocystis jirovecii*, PLT: platelet, PR3: proteinase 3, PT: prothrombin time, RBC: red blood cell, SARS-CoV-2: severe acute respiratory syndrome coronavirus 2, T-Bil: total bilirubin, TP: total protein, WBC: white blood cell, γ-GTP: γ-glutamyl transpeptidase.

Parameter	Value	Unit	Normal range
WBC	6520	/μL	3500-9000
Neutro	80.7	%	-
Lympho	12.0	%	-
RBC	2.41	×10^6^/μL	4.27-5.70
Hb	9.6	g/dL	13.5-17.6
PLT	10.5	×10^4^/μL	13.1-36.2
PT	15.7	sec	10-12
PT%	47.5	%	80-
APTT	34.4	sec	25-36
AST	65	U/L	10-35
ALT	19	U/L	5-40
ALP	147	U/L	38-113
γ-GTP	53	U/L	<60
T-Bil	4.65	mg/dL	<1.0
LDH	572	U/L	124-222
TP	7.3	g/dL	6.6-8.1
Alb	2.1	g/dL	3.7-5.2
BUN	28.5	mg/dL	8-20
Cre	0.81	mg/dL	0.65-1.07
Na	138	mEq/L	136-145
K	3.3	mEq/L	3.5-5.0
Cl	99	mEq/L	98-108
BNP	115.2	pg/mL	<18.4
Glucose	152	mg/dL	70-110
HbA1c	5.8	%	4.6-6.2
CRP	6.47	mg/dL	<0.4
KL-6	1230	U/mL	105.3-401.2
β-D-Glucan	183	pg/mL	<20
CMV antigenemia	Negative		Negative
ANA	40	x	<40
Anti-SS-A antibody	Negative		Negative
Anti-CCP antibody	3.4	U/mL	<4.5
MPO-ANCA	1.9	IU/mL	<3.5
PR3-ANCA	3.0	IU/mL	<2.0
Nasopharynx SARS-CoV-2 antigen	0.19	pg/mL	<1.00
Sputum Pj DNA PCR	Positive		Negative

**Figure 1:**
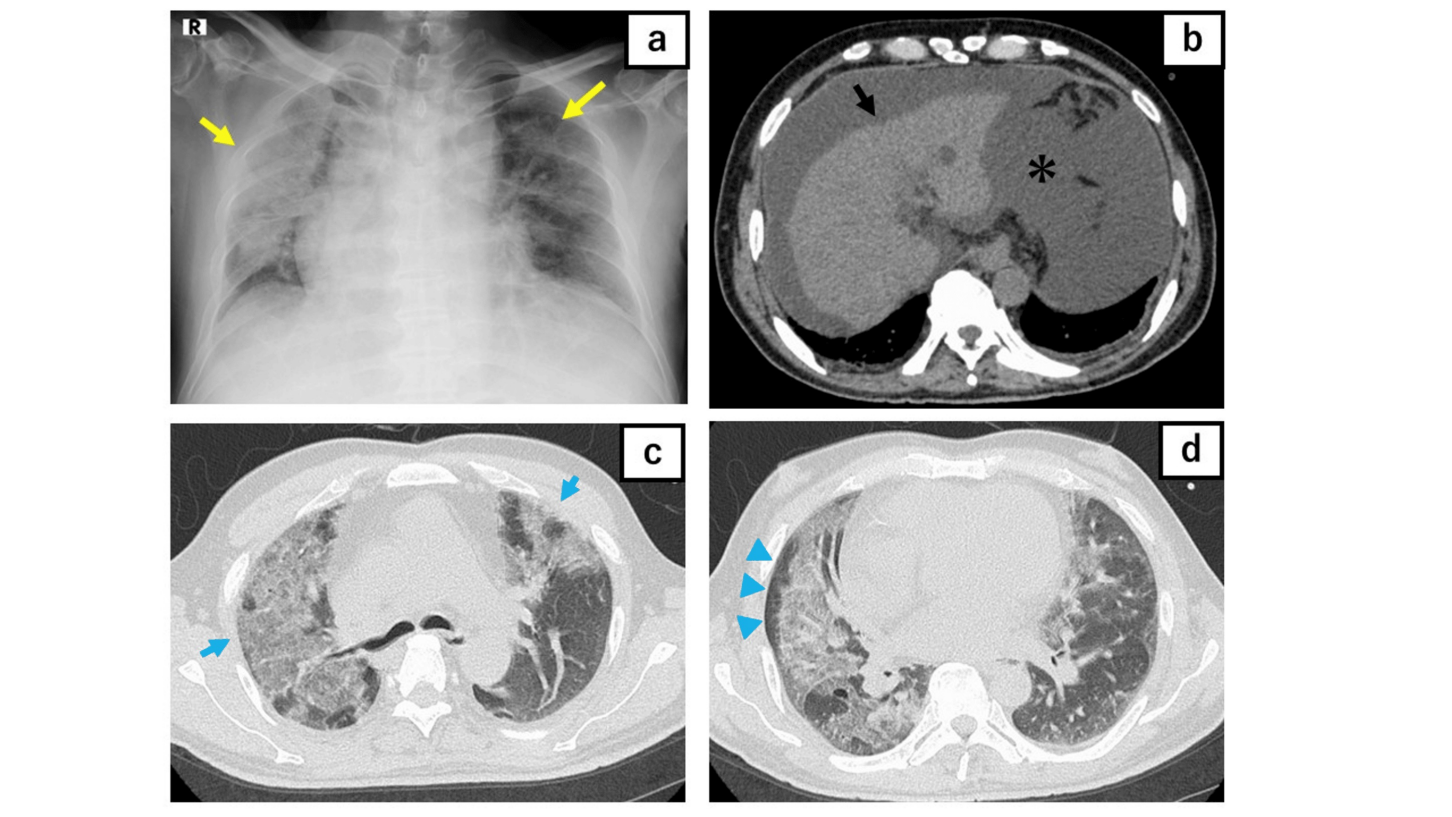
X-ray and CT images A chest X-ray showed bilateral GGOs and consolidation (yellow arrow) (a). A non-contrast abdominal CT scan showed ascites (asterisk) and liver atrophy (black arrow) (b). A chest CT scan demonstrated diffuse GGOs with a mosaic pattern (blue arrow) with subpleural sparing (blue arrowhead) (c, d).

A chest X-ray demonstrated bilateral ground-glass opacities (GGOs) and consolidation, predominantly in the right lung (Figure 1). A subsequent chest CT scan showed diffuse GGOs with a mosaic pattern, also more prominent in the right lung, with evidence of subpleural sparing. Abdominal CT showed ascites and liver atrophy.

The patient was admitted due to respiratory failure and extensive pulmonary infiltrates. Empirical antibiotic therapy with meropenem and levofloxacin was initiated for suspected bacterial pneumonia. However, his respiratory condition worsened over the following day. Considering the possibility of non-infectious pneumonitis such as acute interstitial pneumonia, he received steroid pulse therapy with methylprednisolone for three days.

On the fourth day of hospitalization, a serum β-D-glucan test, drawn at admission, returned with a markedly elevated level. The blood cultures taken at the time of admission were negative. Concurrently, there was no clinically significant increase in various autoantibodies, and there were no physical findings suggestive of collagen disease. Based on the imaging findings and the high β-D-glucan level, PCP was strongly suspected. Treatment with trimethoprim-sulfamethoxazole (TMP-SMX) was initiated at a therapeutic dose (720 mg/day of trimethoprim, about 11.5 mg/kg body weight/day). Prednisolone (PSL) at 30 mg/day was continued as adjunctive therapy for PCP with respiratory failure. The diagnosis of PCP was subsequently confirmed by a positive polymerase chain reaction test for *Pneumocystis jirovecii* (Pj) in a sputum sample. The serum HIV antigen/antibody test was negative.

The patient's respiratory failure showed improvement with PCP treatment; therefore, PSL was gradually tapered and stopped on day 16. Although the patient was treated with levofloxacin for five days and meropenem for nine days on suspicion of a mixed bacterial infection, sputum culture revealed no significant bacterial growth, and the antibiotic therapy was considered to have had no effect on the clinical course. On the other hand, his ascites, related to liver cirrhosis, worsened and remained poorly controlled despite paracentesis and increased diuretics. His general status continued to decline. Although tube feeding was proposed due to poor oral intake, the patient refused. Furthermore, TMP-SMX was discontinued on its 13th day due to the development of progressive renal dysfunction (serum creatinine at 1.98 mg/dL). As the patient's respiratory status remained stable, treatment for PCP with alternative agents was not initiated.

On day 18, the patient experienced an episode of overt aspiration, leading to shock and a recurrence of respiratory failure. Norepinephrine was needed to maintain his blood pressure. Piperacillin-tazobactam was initiated for suspected aspiration pneumonia. However, *Candida krusei* was detected in blood cultures the following day. Despite starting micafungin, his hemodynamic status did not improve. He was refractory to treatment and passed away on day 21.

## Discussion

Liver cirrhosis may not be widely recognized as an independent risk factor for PCP. However, there are several previous case reports (Table 2) [3-8].

**Table 2:** Case reports of PCP in LC patients without immunosuppressive therapy DM: diabetes mellitus, LC: liver cirrhosis, HBV: hepatits B virus, HCV: hepatitis C virus, N/A: not available, PCP: pneumocytis pneumonia, TMP/SMX: trimethoprim/sulfamethoxazole

First author [citation]	Age/Gender	Cause of LC	Treatment	Outcome
Meyers et al. [3]	59/M	Alcohol	Clindamycin + primaquine	Death
Akhter and Pradeep [4]	67/F	HCV	N/A	Survival
Faria et al. [5]	N/A	Alcohol	TMP/SMX	Death
Krier et al. [6]	36/M	Alcohol	TMP/SMX	Survival
Hadfield et al. [7]	63/M	Alcohol	TMP/SMX	Death
Franceshini et al. [8]	56/M	Alcohol	TMP/SMX	Death
	81/M	Metabolic	N/A	Death
	52/M	Alcohol, metabolic	TMP/SMX, clindamycin + primaquine	Death
	66/F	HBV, alcohol	N/A	Death
	34/M	alcohol	TMP/SMX	Survival

Indeed, liver cirrhosis itself is known to cause immune dysfunction [2]. CD4-positive T cells play a crucial role in the immune response against Pj, and a decrease in these lymphocyte counts has been documented in patients with liver cirrhosis [9]. Furthermore, cirrhosis is reported to reduce both the number and function of various immune cells, including not only T cells but also B cells and macrophages [2]. Since the immune response to Pj relies on the complex interplay of these cells [1], the immunosuppression associated with liver cirrhosis can be considered a risk factor for PCP.

Our patient had advanced liver cirrhosis with a Child-Pugh score of 12, which likely induced an immunosuppressed state. While the total blood lymphocyte count was relatively maintained, a decrease or functional impairment in critical subsets, such as CD4-positive cells, may have contributed to the onset of PCP. A limitation of this report is the lack of a detailed evaluation of the patient's immune competence, including serum IgG and CD4 fractions. The estimated serum immunoglobulin level, based on the difference between serum total protein (7.3 g/dL) and albumin (2.1 g/dL), is relatively high, which is thought to be due to the effects of cirrhosis [10]. However, the development of candidemia further supports the presence of severe immunosuppression. Additionally, the patient's malnutrition during hospitalization due to the loss of appetite might have exacerbated the decline in immune function [11, 12]. Glucocorticoids were used to treat PCP, but only for a short period of time and at moderate doses, and its contribution was likely limited.

TMP-SMX was initiated in this patient at a comparatively low dose of trimethoprim (11.5 mg/kg/day), a decision influenced by the concurrent diagnosis of liver cirrhosis. The appropriate dosage of TMP-SMX therapy for the management of PCP has been a topic of recent discussion, and emerging evidence suggests that lower-dose regimens may offer comparable efficacy with a reduced incidence of adverse events [13]. Therefore, we believe the dose used in this patient was appropriate. Although treatment was discontinued prematurely due to adverse effects, the PCP was effectively controlled. Further investigation is warranted to determine the optimal dose for PCP treatment in patients with liver cirrhosis.

Our literature search, including conference abstracts, identified 10 reported cases of PCP in patients with liver cirrhosis who had no history of immunosuppressive therapy and HIV infection (Table 2). The median age in these cases was 59 years, with many having alcohol-related liver disease as the underlying cause. Notably, seven of these 10 patients died, indicating a poor prognosis for this population. Data on the direct causes of death are limited in these cases. However, Franceschini et al. reported esophageal variceal bleeding, multiple organ failure due to sepsis, and concomitant aspergillosis as causes of death [8]. In our case, although the PCP itself tended to improve, the subsequent candidemia was considered the direct cause of death. To the best of our knowledge, this is the first report of secondary candidemia in the context of PCP in a patient with liver cirrhosis. As previously stated, advanced liver cirrhosis creates an immunodeficient state. Therefore, in patients with liver cirrhosis, particularly those who develop PCP, careful monitoring for concomitant other opportunistic infections is crucial.

## Conclusions

This case highlights PCP occurring in a patient with advanced liver cirrhosis. Although the PCP was manageable, the patient ultimately died from a subsequent infection. This case suggests that liver cirrhosis itself can serve as a risk factor for PCP and may be associated with a poor prognosis, especially in the setting of secondary opportunistic infections. Consequently, PCP should be considered in the differential diagnosis when patients with liver cirrhosis present with respiratory failure. Further research is warranted to establish optimal treatment strategies and to clarify the need for prophylaxis for PCP in patients with liver cirrhosis.
